# Gender and Age-Related Differences in Bilateral Lower Extremity Mechanics during Treadmill Running

**DOI:** 10.1371/journal.pone.0105246

**Published:** 2014-08-19

**Authors:** Angkoon Phinyomark, Blayne A. Hettinga, Sean T. Osis, Reed Ferber

**Affiliations:** 1 Faculty of Kinesiology, University of Calgary, Calgary, Alberta, Canada; 2 Faculty of Nursing, University of Calgary, Calgary, Alberta, Canada; 3 Running Injury Clinic, Calgary, Alberta, Canada; Delft University of Technology (TUDelft), Netherlands

## Abstract

Female runners have a two-fold risk of sustaining certain running-related injuries as compared to their male counterparts. Thus, a comprehensive understanding of the sex-related differences in running kinematics is necessary. However, previous studies have either used discrete time point variables and inferential statistics and/or relatively small subject numbers. Therefore, the first purpose of this study was to use a principal component analysis (PCA) method along with a support vector machine (SVM) classifier to examine the differences in running gait kinematics between female and male runners across a large sample of the running population as well as between two age-specific sub-groups. Bilateral 3-dimensional lower extremity gait kinematic data were collected during treadmill running. Data were analysed on the complete sample (*n* = 483: female 263, male 220), a younger subject group (*n* = 56), and an older subject group (*n* = 51). The PC scores were first sorted by the percentage of variance explained and we also employed a novel approach wherein PCs were sorted based on between-gender statistical effect sizes. An SVM was used to determine if the sex and age conditions were separable and classifiable based on the PCA. Forty PCs explained 84.74% of the variance in the data and an SVM classification accuracy of 86.34% was found between female and male runners. Classification accuracies between genders for younger subjects were higher than a subgroup of older runners. The observed interactions between age and gender suggest these factors must be considered together when trying to create homogenous sub-groups for research purposes.

## Introduction

Female runners have a two-fold risk of sustaining certain running-related injuries such as patellofemoral pain syndrome, iliotibial band syndrome, and tibial stress fractures as compared to their male counterparts [1]. Furthermore, it has been reported that female runners exhibit different running kinematic waveform patterns and greater discrete joint angles, which have been postulated to contribute towards greater injury risk [2]–[8]. Traditionally, male and female running patterns have been analysed using discrete time point variables, such as peak angles and angles at touchdown and toe-off, together with inferential statistics, such as the t-test and analysis of variance (ANOVA) [2]–[4]. More recently, pattern recognition methods have been applied in this area, and have achieved good classification performance [5], [6], particularly in combination with a principal component analysis (PCA) approach and a support vector machine (SVM) classifier [7]–[9]. Nigg et al. [7], for example, reported a classification rate of 86.6% between male and female runners using the first 20 principal components (PCs) and an SVM with a linear kernel. However, these previous studies have limitations in terms of the relatively narrow range of age groups sampled (e.g. 24.5±2.5 years for males and 24.6±1.0 years for females [3]), small sample sizes used for classification (e.g. 10 [10], 20 [3], [8], 34 [4], and 40 [2] subjects in total), and many have only measured single-limb lower extremity gait mechanics. Therefore, a comprehensive understanding of the differences in running kinematics between male and female runners may help explain differences in injury patterns between the two populations. To the best of our knowledge, an investigation of bilateral gait measures from a large sample (i.e. several hundred runners) of recreational and competitive runners from both sexes and across a wide distribution of ages has never been completed.

Age-related changes in running kinematic patterns have also been widely reported between younger and older runners [11]–[13]. Specifically, Bus [11] and Fukuchi and Duarte [12] reported significant differences in knee flexion/extension range of motion angle and angle at touchdown between younger and older male runners while no significant differences in peak knee internal rotation and peak rearfoot eversion between the groups were found. On the other hand, Lilley et al. [13] reported significantly increased peak knee internal rotation and peak ankle eversion for an older female group compared to a younger group. One reason for the discrepancy between the results of Bus [11] and Fukuchi and Duarte [12] and that of Lilley et al. [13] may be due to the fact that the gender-specific differences in younger and older runners were not investigated. Thus, a better understanding of whether interactions exist between gender and age, that may affect running kinematics in population sub-groups, is warranted.

Therefore, the first purpose of this study was to examine the differences in running gait kinematics between female and male runners across a large sample of the running population. The second purpose of this study was to examine gender-based differences in kinematics for younger and older age-specific subgroups. Based on results from previous studies, it was hypothesized that: 1) gender-specific kinematic patterns for the whole sample are classifiable with at least 80% accuracy using a PCA approach with an SVM classifier; and 2) different, yet similarly classifiable gender-specific patterns exist for age-specific subgroups.

## Materials and Methods

### Subjects

Four hundred eighty-three recreational and competitive runners participated in this study (Table 1). All were patients who participated in either clinical or research activities at the Running Injury Clinic and all gave informed consent. There were no exclusion criteria based on pain or injury and some participants were pain-free at the time of testing (*n* = 120) while others were experiencing a lower extremity running-related injury (*n* = 363) at the time of testing. However, these injured participants did not experience any pain during treadmill running or the testing procedure and the variables of interest were normally distributed and met the criterion for normal and symmetrical skewness and kurtosis regardless of injury-status. The University of Calgary's Conjoint Health Research Ethics Board (CHREB) approved the collection of the data (Ethics IDs: E–21705, E–22194, E–24339). CHREB approved the consent procedure and written informed consent document. Prior to collecting the data, all participants provide their written informed consent to participate. The storage of the data in the research database and the subsequent approval to analyze the data within the database was also approved by CHREB (Ethics ID E–24519) and all data were identified by a number only and only data related to date of birth, gender, injury status, and athletic history were stored along with all biomechanical data. No personal data, nor any information that could lead to identifying the participant were stored. After the each participant provided their written informed consent, a copy of the informed consent was provided to each participant and also stored in a locked file cabinet.

**Table 1:** Demographic characteristics of study population (mean and (SD)) for general group and two specific subgroups.

Gender	Group	Number of subjects	Age (years)	Height (cm)	Mass (kg)
Male	General subjects (18–72 years)	220	42.1 (11.2)	178.3 (6.9)	79.0 (10.4)
	Young subjects (18–26 years)	16	23.1 (2.6)	181.0 (8.2)	74.1 (9.9)
	Elderly subjects (55–72 years)	34	59.6 (3.7)	178.3 (6.8)	80.5 (10.8)
Female	General subjects (18–72 years)	263	39.3 (11.9)	166.6 (8.3)	64.3 (10.3)
	Young subjects (18–26 years)	40	22.0 (2.9)	166.7 (6.7)	61.2 (6.9)
	Elderly subjects (55–72 years)	17	58.0 (2.1)	162.0 (5.6)	63.6 (8.9)

### Data collection

Eight high-speed digital video cameras (MX3/Nexus, Vicon, Oxford, UK) were used to film treadmill-running at either 120 Hz or 200 Hz. To perform a 3-dimensional (3D) kinematic analysis of running gait, an anatomical model of each subject was constructed based on anatomical marker data collected during a static trial. Spherical retro-reflective markers (9 mm diameter, Mocap Solutions, Huntington Beach, USA) were placed over anatomical landmarks located by palpation and in the same manner described by Pohl et al. [14]. Anatomical markers were placed on the following landmarks: 1^st^ and 5^th^ metatarsal heads; medial and lateral malleoli; medial and lateral femoral condyles; greater trochanter (bilateral); anterior superior iliac spine (ASIS) (bilateral); iliac crest (bilateral). For tracking motion trials, technical marker clusters were placed on the pelvis, and bilateral thigh and shank. A rigid shell with three markers was placed over the sacrum with the two superior markers at the level of the posterior superior iliac spines (PSIS), and rigid shells with four markers were attached to shank and thigh. Technical markers for the foot were placed on the posterior aspect of the shoe: two markers were vertically aligned on the posterior heel counter with a third marker placed laterally. Each participant wore the same shoes (Pegasus, Nike, Beaverton, USA) in order to standardize the footwear condition.

Following placement of all the anatomical and segment markers, the subject was asked to stand on a motorized treadmill instrumented with strain gauges (Bertec Corporation, Columbus, OH, USA) for a static trial. Standing position was controlled using a graphic template placed on the treadmill with their feet positioned 0.3 m apart and pointing straight ahead. Once the feet were placed in the standardized position, the subject was asked to cross their arms over the chest and stand still while one-second of marker location data were recorded. Upon completion of the static trial, the markers on the anatomical landmarks were removed. These markers were not required for a movement trial, and were removed to allow the subject to move less encumbered. The subjects were instructed to warm-up on the treadmill before data were collected for 2–3 minutes and then they ran on the treadmill at a comfortable self-selected pace, between 2.23–3.35 m/s, for 20 seconds in which approximately 30–40 consecutive running strides were collected for processing and analysis.

### Data processing

Kinematic joint angles were calculated using 3D GAIT custom software (Gait Analysis Systems Inc., Calgary, Alberta, Canada) and were analyzed for the stance phase of gait and normalized to 101 data points. Stance and swing phases were defined as initial ground contact to toe-off with initial contact identified as the point in time when the superior calcaneal marker moved from a positive to a negative velocity in the vertical direction, and toe-off was defined when the peak knee extension occurs [15]. For all three planes of motion, and for each of the 6 lower extremity joints, 4 discrete variables of interest were selected based on previous studies [2], [4], [12] consisting of angle at touchdown, peak maximum, peak minimum, and angle at toe-off which resulted in 72 discrete variables of interest used in the PCA. Additionally, these four discrete time points approximated the shape of the kinematic waveform.

### Data analysis

After data analysis for each subject was complete, the discrete variables and demographic data were stored in a relational database using custom MATLAB (The Mathworks, Natick, MA, USA) and MySQL (Oracle, Redwood, CA, USA) code. At a later time point, bulk data were then extracted from the database via MATLAB/MySQL query, and further processing of the data for the purpose of classification was performed in MATLAB on selected variables of interest.

Data were analysed in three groups: a complete sample group (*n* = 483), a younger subject group (*n* = 56) aged 18–26 years and an older subject group (*n* = 51) aged 55–72 years. It should be noted that age range of both groups was defined based on previous literature which reported the significant differences in running kinematics between younger and older groups, for instance, younger-aged (20–35 years) runners and older-aged (55-65 years) runners in the study of Bus [11]. Additionally, Nigg et al. [16] and Lilley et al. [13] reported that changes in gait kinematics begin around 40 years of age.

For each of the subgroups, an original feature vector was created and used as an input for the PCA [17], [18], using an unsupervised learning method. The 72 discrete variables comprised the columns and the 483, 56, and 51 subjects comprised the rows of the matrix for the general subject group, younger, and older subject subgroups (*X*_483×72_, *X*_56×72_, *X*_51×72_), respectively. PCA uses an orthogonal transformation to convert a set of possibly correlated variables into a set of linearly uncorrelated variables and tries to account for as much of the variability in the original data as possible in the first components. The first step in the PCA was to standardize the original feature vector, then transform into PCs using an eigenvector decomposition method on the input's covariance matrix. The eigenvectors (*V*_72×72_, *V*_72×55_, *V*_72×50_) and eigenvalues (*L*_1×72_, *L*_1×55_, *L*_1×50_) were produced and used to compute the PC scores (*Z*_483×72_, *Z*_56×55_, *Z*_51×50_), by multiplying the standardized feature matrix by the eigenvector matrix. The PC scores were first sorted by the percentage of variance explained by each. However, sample variance detected by PCA does not necessarily reflect variation between genders, and therefore, may not be indicative of the differences between male and female runners. Consequently, PCs were also sorted based on between-gender effect sizes, which were calculated using Hedges's *g* [19].

Finally, an SVM supervised learning method was used to determine if the sex and age conditions were separable and classifiable based on the PCs [20]. The binary SVM classifier constructed a set of the optimal hyperplanes in high-dimensional space, which represents the largest margin, or distance between the support vectors, or the nearest training data points of the two classes. In the case that all training points cannot be separated by the hyperplane, a soft margin method was used to construct a hyperplane that separates the training data points [21]. A soft margin parameter *c* was set at 1 based on the methods reported by Fukuchi et al. [22]. A ten-fold cross validation method was applied to obtain classification rates from the SVM classifier. All PC data were randomly partitioned into 10 equally sized sub-datasets and a single sub-dataset was retained as testing data while the remaining 9 sub-datasets were used as training data for the classification model. The cross-validation process was then repeated 10 times, and a single classification rate was computed by averaging from 10 results. Two-sample *t*-tests were used to test for statistically significant differences (*p*<0.05). The resulting *p*-values were adjusted using a Holm-Bonferroni method to maintain a family-wise alpha of 0.05 for tests on all PCs and discrete variables.

## Results

### Gender difference in general population

**Figure 1:**
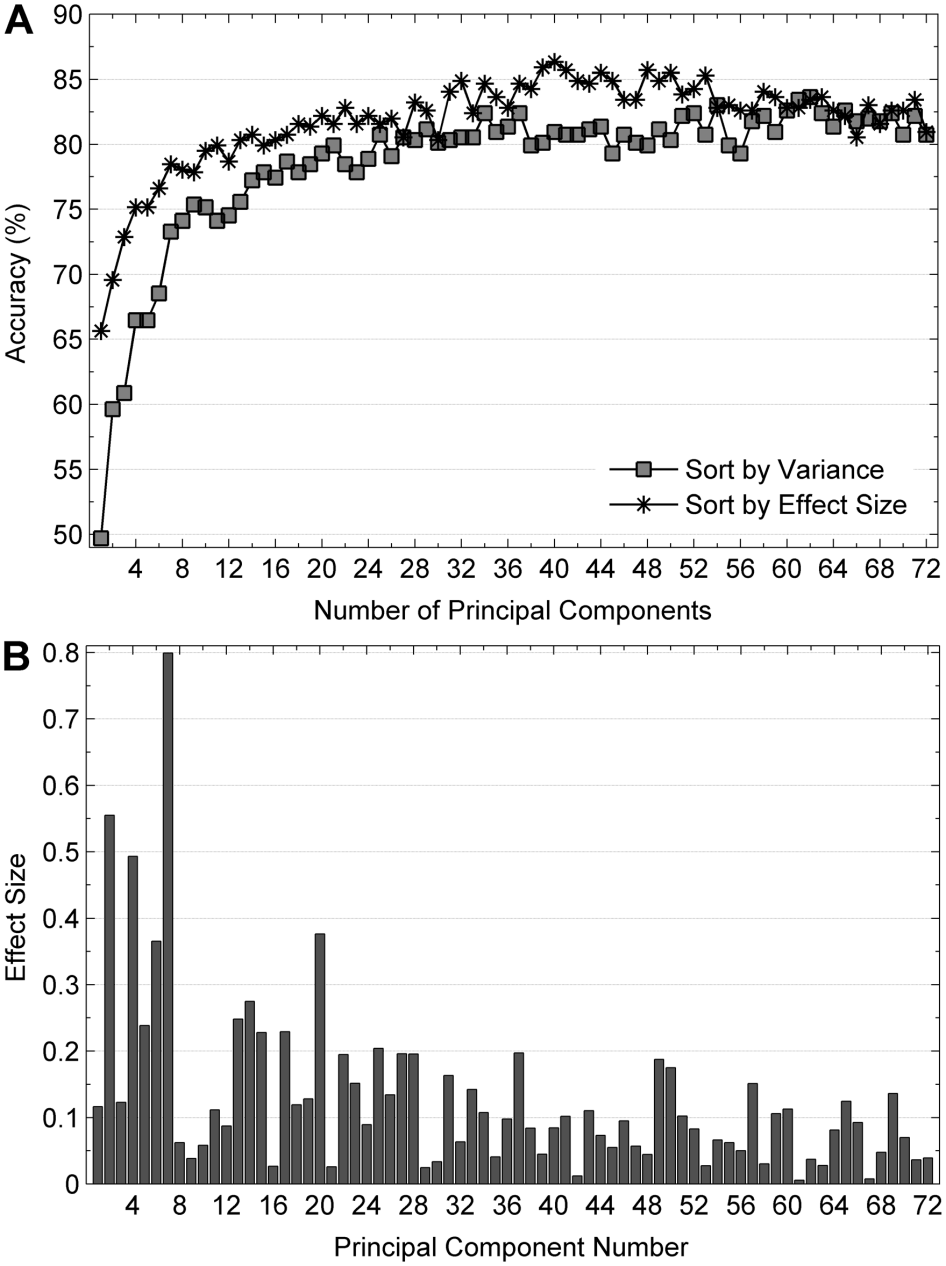
Classification rates and effect sizes for gender difference in general subject group. (a) Classification rates for gender difference computed from a support vector machine classifier with a ten-fold cross validation method on PCs sorted by variance explained and effect size for general subject group. (b) Effect sizes of all PCs computed from general subject group for gender difference.

The first 62 PCs explained 99.94% of the variance in the data and the SVM classification accuracy of 83.64% was found between male and female runners. When feature vectors were created based on PC scores sorted by effect size, as opposed to percent variance explained, a classification accuracy of 86.34% was found using 40 PCs, which explained 84.74% of the variance in the data. The remaining PCs, which were not used in an optimized feature vector, had effect sizes less than 0.09. Mean classification accuracies for gender for all PCs are presented in Fig. 1(a) and the effect size for each of the 72 PC scores is shown in Fig. 1(b). Only PC 7 showed a large effect size (0.80) while PC 2 and 4 showed a medium effect size (0.56 and 0.49) for the projection difference between the 220 male and 263 female subjects. PC 7, 2, and 4 explained 26.40% of the variance in the data.

Forty-seven of the 72 discrete biomechanical variables correlated with PC 7 at a significance level of *p*<0.01, and 34 variables were significant at *p*<0.0001. In addition, 52 (*p*<0.01) and 36 (*p*<0.001) variables were significantly correlated with PC 2, and 55 (*p*<0.01) and 43 (*p*<0.001) variables were significantly correlated with PC 4. Statistical analysis of original discrete variables (Table 2) showed that, in the frontal plane, female runners demonstrated greater maximum and minimum peak hip adduction and knee abduction, greater hip adduction at touchdown, and greater hip adduction and knee abduction at toe-off compared to males (*p*<0.01). Also in the transverse plane, female runners exhibited greater external rotation of the femur at touchdown and maximum peak (*p*<0.01). Conversely, in the frontal plane, female runners exhibited reduced peak ankle eversion compared to males. In the sagittal plane, female runners exhibited reduced minimum peak knee flexion, peak ankle dorsiflexion, and knee flexion at toe-off compared to males (*p*<0.01). Frontal plane hip angles were moderately related to PC 7, 2, and 4 while frontal plane knee angles for both legs were strongly related to PC 2. It is also interesting to note that no correlations and/or significant differences in sagittal plane knee and hip kinematic variables were observed between male and female runners using a PCA approach. All correlation coefficients between PC 7, 2, and 4 and the significant discrete biomechanical variables are shown in Table 3.

**Table 2:** Comparisons of the discrete biomechanical variables (mean and (SD)) between male and female runners for general group.

Joint	Plane of motion	Variable of interest	Left lower limb		Right lower limb	
			Male	Female	Male	Female
Hip	Frontal	Maximum peak a ^,^ b	9.50 (4.30)	11.45 (4.03)	8.00 (3.62)	10.63 (3.74)
		Minimum peak a	−0.15 (3.49)	1.20 (3.79)	−0.91 (3.70)	−0.14 (3.89)
		At toe-off a ^,^ b	3.06 (3.35)	5.97 (3.06)	4.05 (3.14)	6.72 (3.36)
	Transverse	At touchdown a ^,^ b	8.87 (12.03)	12.87 (6.90)	11.75 (6.57)	14.85 (6.43)
		Maximum peak a ^,^ b	10.34 (11.99)	14.15 (6.86)	13.32 (6.31)	16.49 (6.26)
Knee	Frontal	At touchdown a ^,^ b	−5.59 (3.72)	−8.01 (3.41)	−6.31 (3.53)	−8.59 (3.49)
		Maximum peak a ^,^ b	−8.55 (5.18)	−10.75 (4.35)	−9.94 (4.79)	−11.95 (4.50)
		Minimum peak a ^,^ b	−2.99 (4.67)	−5.46 (3.89)	−4.33 (4.05)	−6.61 (3.78)
		At toe-off a ^,^ b	−5.31 (3.95)	−7.70 (3.37)	−6.11 (3.59)	−8.67 (3.39)
	Sagittal	Minimum peak a	14.88 (5.43)	12.66 (5.45)	13.26 (4.78)	12.02 (5.02)
		At toe-off a ^,^ b	17.28 (6.31)	14.25 (6.37)	15.60 (5.69)	13.49 (5.76)
Ankle	Frontal	Minimum peak a ^,^ b	−7.30 (3.18)	−5.54 (3.13)	−7.13 (3.07)	−5.64 (3.16)
	Sagittal	Minimum peak b	−23.37 (2.72)	−22.50 (2.73)	−22.92 (2.81)	−21.99 (2.93)

a Significant gender difference for left lower limb (adjusted *p*-value <0.01).

b Significant gender difference for right lower limb (adjusted *p*-value <0.01).

**Table 3:** Correlation coefficients between three significant PCs: 7, 2, and 4, and the significant original discrete variables for general group.

Joint	Plane of motion	Variable of interest	Left lower limb			Right lower limb		
			PC 7	PC 2	PC 4	PC 7	PC 2	PC 4
Hip	Frontal	Maximum peak	*0.41*	0.32	0.03	0.10	0.15	*0.40*
		Minimum peak	0.26	0.16	0.06	*0.37*	*0.40*	*0.36*
		At toe-off	*0.47*	0.27	*0.41*	*0.43*	*0.38*	*0.42*
	Transverse	At touchdown	0.22	0.07	0.27	0.32	0.17	0.16
		Maximum peak	0.22	0.08	0.27	0.08	0.06	0.01
Knee	Frontal	At touchdown	0.29	*0.70*	0.10	0.22	*0.72*	0.25
		Maximum peak	0.21	*0.75*	0.12	0.09	*0.83*	0.01
		Minimum peak	0.20	*0.78*	0.01	0.12	*0.77*	0.25
		At toe-off	0.23	*0.67*	0.20	0.14	*0.70*	*0.38*
	Sagittal	Minimum peak	0.18	0.05	0.09	0.20	0.07	0.08
		At toe-off	0.26	0.05	0.15	0.29	0.09	0.15
Ankle	Frontal	Minimum peak	0.17	0.13	*0.45*	*0.47*	0.31	0.33
	Sagittal	Minimum peak	0.09	0.05	0.29	0.12	0.16	0.30

Italic number shows a moderate correlation (*r*≥0.36) and bold number shows a strong correlation (*r*>0.67) [23].

### Age effects on the gender difference

**Figure 2:**
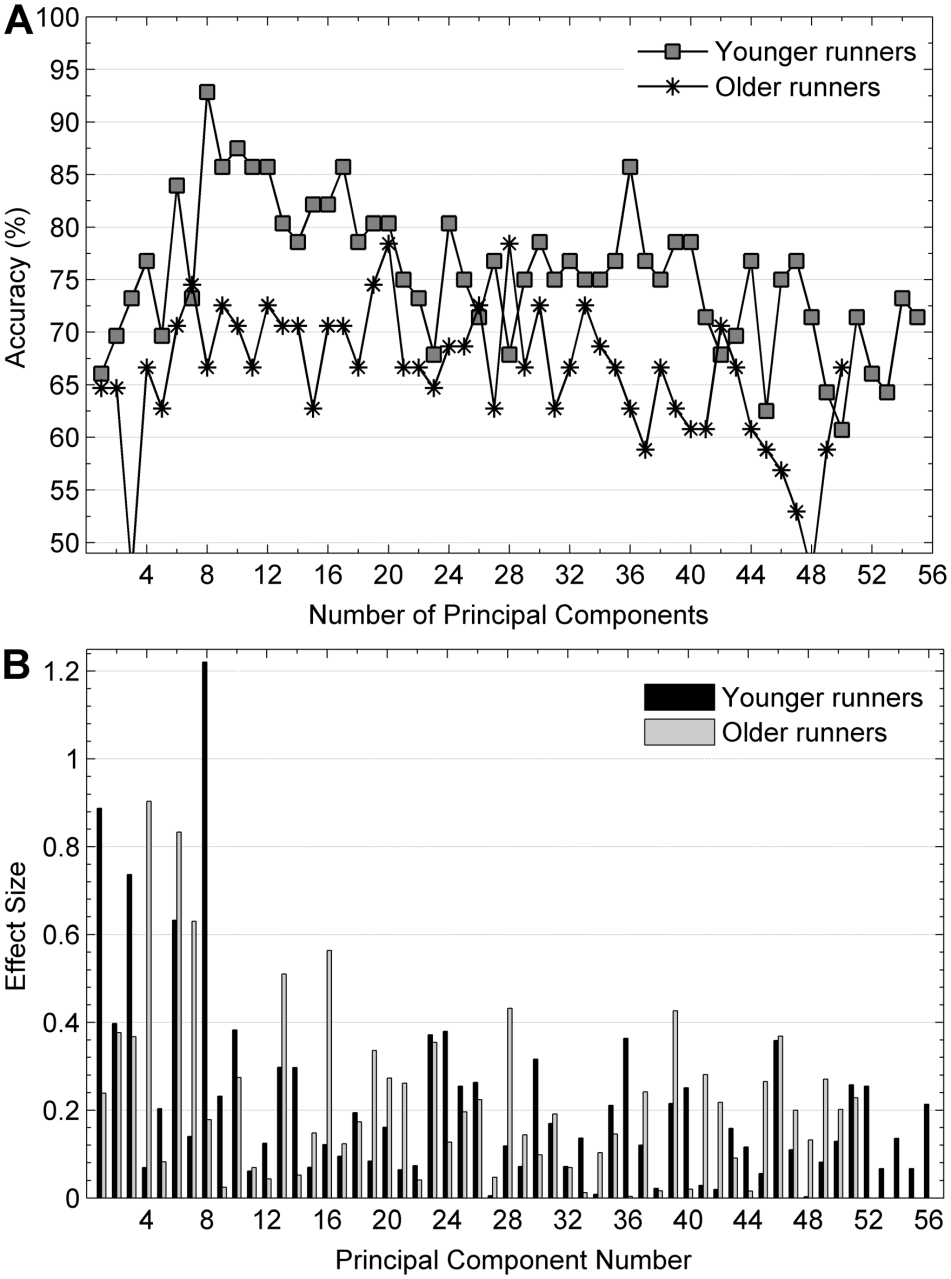
Classification rates and effect sizes for gender difference in younger and older subject subgroups. (a) Classification rates for gender difference computed from a support vector machine classifier with a ten-fold cross validation method on PCs sorted by effect size for younger and older subject subgroups. (b) Effect sizes of all PCs computed from younger and older subject subgroups for gender difference.

Classification accuracies between genders in a subgroup of 56 younger subjects were significantly higher than a subgroup of 51 older subjects (*p*<0.01), as can be seen in Fig. 2(a). Specifically, classification accuracies of 92.86% and 78.43% were found using the first 8 and 20 PCs, which explained 78.52% and 95.66% of the variance in the data for the younger and older subject subgroups, respectively. When PC score vectors were created based on effect size, males and females could be separated with 100% classification accuracy using a linear SVM ten-fold cross-validation method with the 15 and 34 PCs explaining 64.80% and 82.91% of the variance in the data for the younger and older subject subgroups, respectively. The classification rate decreased when PC scores with effect sizes less than or equal to 0.07 were added into feature vectors for both subgroups. PC 8 and 1 showed a large effect size (1.22 and 0.89) and PC 3 and 6 showed a medium effect size (0.74 and 0.63) for the projection difference between male and female younger subjects, while PC 4 and 6 showed a large effect size (0.90 and 0.83) and PC 7, 16, and 13 showed a medium effect size (0.63, 0.56, and 0.51) for the older subjects. The effect sizes for all the 55 PC scores for younger group and the 50 PC scores for older group are shown in Fig. 2(b).

Tables 4–7 present a summary of the significant discrete biomechanical variables between male and female runners, and the correlation coefficients between the PCs and the discrete biomechanical variables for younger and older subgroups, respectively. Both younger and older female runners demonstrated greater hip adduction at toe-off (*p*<0.05) and this angle was also related to the PCs that provided the most separability. Younger female runners demonstrated reduced peak ankle dorsiflexion, reduced knee flexion and internal rotation of the femur, and greater external rotation of the femur at all time points throughout stance phase compared to younger male runners (*p*<0.05). Transverse plane hip angles were moderately related to PC 1 and 3 while sagittal plane knee angles were strongly related to PC 1 for the younger subject subgroup. In addition, greater knee abduction at all time points and a lower peak ankle eversion were observed for older female runners compared to older males (*p*<0.05).

**Table 4:** Comparisons of the discrete biomechanical variables (mean and (SD)) between male and female runners for younger group.

Joint	Plane of motion	Variable of interest	Left lower limb		Right lower limb	
			Male	Female	Male	Female
Hip	Frontal	At toe-off a ^,^ b	0.69 (2.70)	5.25 (2.47)	2.80 (3.13)	6.46 (2.90)
	Transverse	At touchdown a	7.10 (7.56)	14.86 (5.95)	12.82 (7.41)	14.89 (6.59)
		Maximum peak a	8.32 (7.98)	16.32 (6.17)	14.08 (7.35)	17.28 (6.36)
		Minimum peak a	−8.26 (6.05)	−1.29 (5.94)	−1.89 (5.90)	−0.03 (7.81)
		At toe-off a	−7.94 (5.93)	−1.27 (5.95)	−1.51 (5.87)	−0.02 (7.81)
Knee	Sagittal	At touchdown b	18.19 (2.99)	14.86 (4.97)	17.24 (3.46)	12.96 (5.13)
		Maximum peak a	48.19 (5.61)	42.50 (6.20)	46.45 (5.05)	41.27 (6.27)
		Minimum peak b	15.65 (4.09)	11.91 (5.22)	14.83 (4.03)	10.58 (4.48)
		At toe-off b	17.81 (5.54)	13.21 (6.27)	17.11 (4.75)	12.06 (4.79)
Ankle	Sagittal	Minimum peak a ^,^ b	−24.65 (2.55)	−22.25 (2.42)	−24.00 (1.98)	−21.24 (2.82)

a Significant gender difference for left lower limb (adjusted *p*-value <0.05).

b Significant gender difference for right lower limb (adjusted *p*-value <0.05).

**Table 5:** Correlation coefficients between three significant PCs: 8, 1, and 3, and the significant original discrete variables for younger group.

Joint	Plane of motion	Variable of interest	Left lower limb			Right lower limb		
			PC 8	PC 1	PC 3	PC 8	PC 1	PC 3
Hip	Frontal	At toe-off	*0.45*	*0.36*	*0.54*	*0.48*	0.10	*0.48*
	Transverse	At touchdown	0.16	*0.62*	*0.45*	0.08	0.32	*0.61*
		Maximum peak	0.16	*0.62*	*0.44*	0.08	0.21	*0.63*
		Minimum peak	0.19	*0.54*	*0.57*	0.11	*0.43*	*0.61*
		At toe-off	0.18	*0.53*	*0.56*	0.10	0.19	*0.62*
Knee	Sagittal	At touchdown	0.20	***0.67***	0.22	0.30	*0.63*	0.12
		Maximum peak	0.23	***0.68***	0.04	0.25	***0.69***	0.10
		Minimum peak	0.18	***0.74***	0.29	0.33	***0.71***	0.26
		At toe-off	0.12	*0.75*	0.26	0.31	***0.72***	0.24
Ankle	Sagittal	Minimum peak	0.07	*0.52*	0.03	0.11	*0.59*	0.01

Italic number shows a moderate correlation (*r*≥0.36) and bold number shows a strong correlation (*r*>0.67).

**Table 6:** Comparisons of the discrete biomechanical variables (mean and (SD)) between male and female runners for older group.

Joint	Plane of motion	Variable of interest	Left lower limb		Right lower limb	
			Male	Female	Male	Female
Hip	Frontal	At toe-off a ^,^ b	4.77 (3.48)	8.40 (2.88)	4.80 (2.98)	7.55 (3.08)
Knee	Frontal	At touchdown a ^,^ b	−5.51 (3.27)	−9.12 (3.59)	−6.46 (3.61)	−9.40 (2.79)
		Maximum peak a	−7.68 (4.36)	−11.82 (5.02)	−9.17 (4.52)	−11.81 (4.74)
		Minimum peak a	−2.78 (4.92)	−7.10 (4.36)	−4.28 (4.63)	−6.81 (4.48)
		At toe-off a	−5.99 (3.81)	−9.30 (4.01)	−6.43 (4.29)	−8.73 (3.23)
Ankle	Frontal	Minimum peak a	−7.30 (2.62)	−4.99 (2.75)	−7.14 (3.52)	−4.74 (3.32)

a Significant gender difference for left lower limb (adjusted *p*-value <0.05).

b Significant gender difference for right lower limb (adjusted *p*-value <0.05).

**Table 7:** Correlation coefficients between three significant PCs: 8, 1, and 3, and the significant original discrete variables for older group.

Joint	Plane of motion	Variable of interest	Left lower limb			Right lower limb		
			PC 4	PC 6	PC 7	PC 4	PC 6	PC 7
Hip	Frontal	At toe-off	*0.53*	*0.47*	0.29	0.13	*0.40*	0.07
Knee	Frontal	At touchdown	0.35	0.27	0.33	0.17	0.31	0.11
		Maximum peak	0.27	0.16	0.33	0.01	0.15	0.18
		Minimum peak	0.17	0.10	0.30	0.10	0.16	0.21
		At toe-off	0.31	0.17	0.35	0.19	0.15	0.16
Ankle	Frontal	Minimum peak	*0.61*	0.10	0.13	*0.49*	0.23	0.30

Italic number shows a moderate correlation (*r*≥0.36).

## Discussion

### Classification accuracy

The primary purpose of this study was to examine the effects of gender on running kinematics in a large sample of the running population. Previous investigations that have utilised a PCA and SVM approach have reported sex-specific classification accuracies between 80%–95% [5], [7], [8]. In support of our hypotheses, and consistent with previous literature, the results of the current study show that a classification accuracy of 86.34% was found across a wide range of female and male runners regardless of other subject-specific differences, injury status, and test conditions including age.

Our results also indicate that higher classification accuracy can be achieved using age-specific subgroups since the amount of between-group variance can be explained using a fewer number of PCs and the effect size of the associated PC scores will subsequently increase. A strength of the current study as compared to previous investigations of sex- and age-specific differences in running gait mechanics, is that prior works have used a relatively narrow sample of the general population, with groups of 5 to 56 subjects for male and female runners [2]–[8], [10]. Moreover, Nigg et al. [7] also presented PC classification data on age-specific sub-groups of male and female runners but only had sample sizes of 10 to 13 subjects per group. The current study improves upon prior literature by increasing the sample size by 4–44 times, and by drawing from a wide range of running participants. The results of this investigation also demonstrate there are interactions between age and gender which affect running kinematics and, consequently, it is strongly recommended that sex and age be considered together when trying to create homogenous sub-groups for research purposes. This approach has the added advantage of classifying gait pattern differences without the need for matched training subject data that would be impractical for automatic recognition systems.

### Selection of components in PCA

Typically, the choice of PCs used in a feature vector is based on the process of plotting eigenvalues according to their size (scree plot), keeping only the PCs whose eigenvalue is larger than the average (>1.0), or keeping the first PCs that explain at least 95% of cumulative variance in the data [7], [8], [24]–[26]. In the current study, a novel method was used, which sorted PCs based on effect size. This method was chosen based on the work of Ferré [27] who suggested that there is no one solution that is suitable for all problems and most rules fail to determine the optimal number of PCs. Since effect size is directly related to the discrimination being considered, it therefore constitutes a context-specific rule that can be applied to the research question in order to obtain PCs that are most appropriate to the specific research purpose [28].

Comparing both methods, maximum accuracy was found when the PCs were sorted by the effect size as opposed to percentage of variance. Although the first three PCs were common to both sorting methods, in order to achieve the maximum classification rate for gender, intermediate- and higher-order PCs were needed to maximize performance of the SVM classifier. For example, it is interesting to note that the highest PC selected and included in an optimized feature vector was PC 69. This result supports the work of Maurer et al. [8], who demonstrated that PCs which best explained differences between male and female runners were within both the lower-order, or basic movement PCs, as well as the higher-order, or subtle movement PCs (PC 10–PC 41). In other words, it can be postulated that the lower PCs have a low effect size for determining gender differences in running kinematics, and could be considered noise in the context of gender discrimination. This supposition could also explain why the maximum classification accuracy was not achieved when the PCs were sorted by more traditional methods such as percent of the variance explained in the data. Future research is needed in this area to better understand the relationship between lower- and higher-order PCs and their usefulness in explaining between-group differences.

### Discrete kinematic variables

The results of the current study suggest that several biomechanical variables had a moderate (*r*≥0.36) and statistically significant (*p*<0.0001) correlation with the discriminatory PCs when determining differences in running gait kinematics across the general population. When assessing differences between female and male runners across the general population, female runners generally demonstrated greater frontal plane hip and knee peak angles and differences in frontal plane hip and knee angles at touchdown and toe-off as compared to their male counterparts. Female runners also exhibited a greater transverse plane hip peak angle and differences in transverse plane hip angles at touchdown as compared to males. Conversely, female runners exhibited a reduced sagittal plane knee peak angle and differences in sagittal plane knee angles at toe-off as compared to males. These results are consistent with previous literature suggesting female runners generally demonstrate greater frontal and transverse plane angles [2]–[4], [7], [8] and reduced sagittal plane knee angles [3] as compared to male runners. However, these results also suggest that the discrimination of running kinematics between male and female runners is a complex classification problem, reflecting relationships amongst many kinematic variables [29]. Therefore, simplistic approaches, such as analyzing several discrete kinematic and/or kinetic variables, and the use inferential statistics are not recommended.

When female and male runners were sub-grouped according to specific age categories, significant differences in sagittal plane knee kinematic variables were observed between male and female younger runners using a PCA approach. Our results are similar to previous studies [7] including Fukuchi et al. [22] who used an SVM classifier and a forward feature selection approach, and reported that the feature containing the most discriminative information was the greater knee flexion excursion angle exhibited by the younger runners as compared to an older cohort. Therefore, it appears that older adult runners, regardless of sex, exhibit reduced sagittal plane joint kinematics as compared to their younger counterparts. These results are similar to previous studies that have suggested age-related biomechanical alterations during gait are a consequence of reduced muscle strength and flexibility; the combined result of sarcopenia and biological aging [30], [31].

Older female runners also exhibited greater knee abduction, at all selected time points, and reduced peak ankle eversion as compared to older male runners. These results suggest that sex-specific frontal plane differences are present regardless of biological ageing. On the other hand, no differences in transverse-plane ankle kinematics and no differences in frontal and sagittal plane hip kinematics were observed between young and elderly runners, findings that are consistent with previous studies [7], [11]–[13], [22]. These results also support the premise that age- and sex-specific kinematic patterns are present and must be accounted for in future research investigations. Therefore, the findings from the present investigation may shed light onto the conflicting results from various gender-based investigations, which all involved different subject sub-groups [2]–[4], [7], [8].

### Limitations

Limitations to the current research study are acknowledged. First, we did not collect ground reaction force and thus kinetic or joint moment information was not included in the analysis. Nigg et al. [7] also used a similar PCA approach with an SVM classifier and also limited their analysis to kinematic variables. Moreover, these authors used a position matrix based on the marker position data for the PCA analysis, and while similar classification accuracy was found as compared to the current study, the clinical relevance of marker position data is questionable. Thus, we chose to use joint kinematic angles to improve the clinical relevance of the results and hopefully shed some light on the disparity in running-related injuries between males and females. Regardless, future studies should also incorporate joint kinetic and ground reaction force data to gain a greater understanding of sex- and age-related differences in running gait biomechanics. Second, we only reported on data derived from the stance phase of running gait. It is possible that useful and discriminative information may also be found during the full stride cycle and we chose to only focus our attention on the stance phase of gait based on previous investigations [7], [8]. Future research should consider analysis of both the swing and stance phases of running gait. Finally, the current study used a large cohort of both non-injured and injured participants. While the injured participants did not experience any pain during treadmill running or the testing procedure, they could have experienced altered gait kinematics as a function of the injury itself. However, the variables of interest were normally distributed for both the injured and the non-injured runners. Thus, the data being analysed were representative of running gait based on the large number and wide range of running participants involved in the current study.

## Conclusions

In conclusion, using a principal component analysis approach, combined with a support vector machine classifier, the present study accurately classified large cohorts of competitive and recreational male and female runners across a wide spectrum of age. To our knowledge the current study improves upon prior literature by increasing the sample size by 4–44 times, and by drawing from a wide range of running participants. When the study population was divided into two age-specific sub-groups, interactions between age and gender were observed suggesting that sex and age must be considered together when trying to create homogenous sub-groups for research purposes.
