# Correction: Gender and Age-Related Differences in Bilateral Lower Extremity Mechanics during Treadmill Running

**DOI:** 10.1371/journal.pone.0116643

**Published:** 2014-12-23

**Authors:** The PLOS ONE Staff

There are errors in the legend for Figure 2, “Classification rates and effect sizes for gender difference in younger and older subject subgroups.” Please see the complete, correct Figure 2 legend here.

There are errors in Table 3. The first four values in the PC2 columns of the Knee row should be bold. The authors have provided the corrected table below.

**Table 3:** Correlation coefficients between three significant PCs: 7, 2, and 4, and the significant original discrete variables for general group.

Joint	Plane of motion	Variable of interest	Left lower limb			Right lower limb		
			PC 7	PC 2	PC 4	PC 7	PC 2	PC 4
Hip	Frontal	Maximum peak	*0.41*	0.32	0.03	0.10	0.15	*0.40*
		Minimum peak	0.26	0.16	0.06	*0.37*	*0.40*	*0.36*
		At toe-off	*0.47*	0.27	*0.41*	*0.43*	*0.38*	*0.42*
	Transverse	At touchdown	0.22	0.07	0.27	0.32	0.17	0.16
		Maximum peak	0.22	0.08	0.27	0.08	0.06	0.01
Knee	Frontal	At touchdown	0.29	***0.70***	0.10	0.22	***0.72***	0.25
		Maximum peak	0.21	***0.75***	0.12	0.09	***0.83***	0.01
		Minimum peak	0.20	***0.78***	0.01	0.12	***0.77***	0.25
		At toe-off	0.23	***0.67***	0.20	0.14	***0.70***	*0.38*
	Sagittal	Minimum peak	0.18	0.05	0.09	0.20	0.07	0.08
		At toe-off	0.26	0.05	0.15	0.29	0.09	0.15
Ankle	Frontal	Minimum peak	0.17	0.13	*0.45*	*0.47*	0.31	0.33
	Sagittal	Minimum peak	0.09	0.05	0.29	0.12	0.16	0.30

Italic number shows a moderate correlation (*r* ≥ 0.36) and bold number shows a strong correlation (*r*> 0.67) [23].

**Figure 2:**
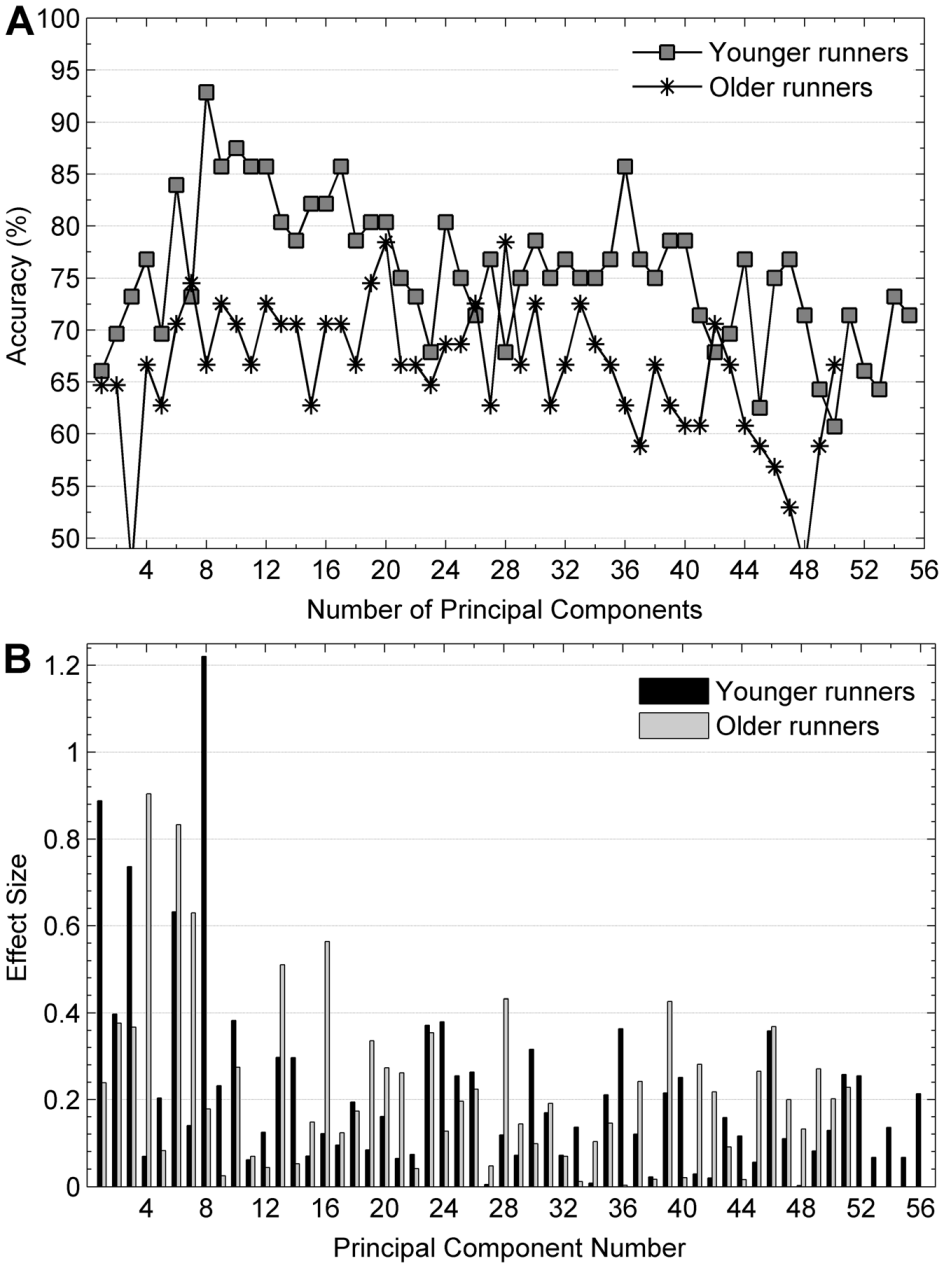
Classification rates and effect sizes for gender difference in younger and older subject subgroups. (a) Classification rates for gender difference computed from a support vector machine classifier with a ten-fold cross validation method on PCs sorted by variance explained for younger and older subject subgroups. (b) Effect sizes of all PCs computed from younger and older subject subgroups for gender difference.
